# An Investigation of the Effect of Exercise on Sleep Disturbances and Fatigue Symptoms in Patients Diagnosed with Primary Brain Tumors: A Systematic Review

**DOI:** 10.3390/neurosci7010014

**Published:** 2026-01-15

**Authors:** Eleftheria Ntalagianni, Eleni Katsouli, Anna Christakou, Dimitrios Chytas, Piergiorgio Lochner, Epameinondas Lyros

**Affiliations:** 1 Department of Physiotherapy, School of Health Sciences, University of Peloponnese, 23100 Sparta, Greece; ellie.ntala@gmail.com (E.N.); elenakatsouli03@gmail.com (E.K.); a.christakou@go.uop.gr (A.C.); dimitrios.chytas@uop.gr (D.C.); 2 Department of Neurology, Saarland University Medical Center, 66421 Homburg, Germany; piergiorgio.lochner@uks.eu

**Keywords:** brain tumor, sleep disorder, fatigue, different type of exercise

## Abstract

Background: Patients with primary central nervous system (CNS) tumors often experience fatigue and sleep disturbances, significantly impacting their quality of life. Exercise has been shown to improve these symptoms in various cancer populations. The aim of this study is to evaluate the effects of different types of exercise on fatigue and sleep in less-investigated CNS tumor patients. Methods: A literature search was conducted in PubMed, Scopus, Cochrane Library, and CINAHL. Eligible randomized and non-randomized studies evaluating exercise interventions in patients diagnosed with primary brain tumors were systematically reviewed, primarily using a narrative synthesis approach. Cancer-related fatigue and sleep-related outcomes were extracted as variables of interest. Where possible [≥2 Randomized Control Trials (RCTs) available for glioma patients], meta-analyses were conducted to assess the overall effects of physical therapy on the above-mentioned outcomes. Results: A total of 15 relevant intervention studies were identified, either RCTs or other types of studies, such as prospective feasibility cohort studies and case studies. A total of 448 participants were enrolled, with the majority diagnosed with glioma. There were single reports on pituitary adenoma after surgery and meningioma patients. In glioma patients, the overall effect of various modality exercise interventions on fatigue was non-significant, reflecting the heterogeneous characteristics of studies with diverse outcomes. However, meta-analysis focusing on combined exercise interventions (aerobic and resistance training) showed a positive effect on reducing fatigue in these patients [Standardized Mean Difference (SMD) = 0.866, *p* = 0.03]. Fatigue in glioma patients may also improve through yoga and Pilates. Aerobic but not strength exercise seems to improve sleep in glioma patients (SMD = 1.14, *p* = 0.02). Sleep quality may also improve through yoga and combined exercise. Conclusions: Certain types of exercise appear to effectively reduce fatigue and improve sleep in patients with CNS tumors. Future, well–controlled, multi-arm, larger-scale studies are necessary to resolve discrepancies, as well as to explore long-term outcomes and define factors influencing individualized exercise responses.

## 1. Introduction

One of the leading causes of death worldwide is cancer [1]. Brain and CNS tumors, although relatively rare, account for about 1.5% of all diagnosed cancers and are associated with high morbidity rates and unfavorable prognosis [2]. Malignant gliomas are a group of primary brain tumors that, in clinical practice, primarily refer to glioblastoma and other high-grade diffuse gliomas. Gliomas are predominantly adult-onset tumors with incidence increasing with age. The overall incidence of gliomas in adults is estimated at approximately 6 per 100,000 person-years [3]. Glioblastoma, the most frequent and most aggressive subtype, has a median age at diagnosis of approximately 65 years and an age-adjusted incidence of about 3.22 per 100,000 [4]. Glioblastoma, accounting for up to 50% of all malignant brain neoplasms, is characterized by rapid progression and a poor prognosis, often leading to death within a short clinical course [5,6,7]. Diffuse gliomas other than glioblastoma are predominantly classified as World Health Organization (WHO) grade 3 or 4 tumors. Oligodendroglioma, a rare subtype of diffuse malignant glioma, occurs primarily in adults and is molecularly distinct from both IDH-mutant astrocytoma and IDH wild-type glioblastoma. It accounts for approximately 5% of all primary intracranial tumors [8,9]. Astrocytomas represent another major category of diffuse malignant gliomas, comprising approximately 60% of all primary brain tumors [10]. IDH-mutant diffuse astrocytomas are classified across grades 2 to 4, with grade 4 being the most aggressive form. Notably, grade 4 IDH-mutant astrocytoma exhibits a better prognosis compared to IDH wild-type glioblastoma, CNS WHO grade 4 [5]. The most common and predominantly benign of all primary CNS tumors are meningiomas, tumors that usually progress slowly. They often have a favorable clinical course, with approximately 20% experiencing local recurrence and/or progressing to a more severe and more aggressive form [11]. Pituitary region tumors constitute the most prevalent subgroup of CNS neoplasms among children, adolescents, and young adults (15–39 years). Pituitary adenomas are benign tumors that most commonly arise in the anterior pituitary gland. The estimated annual incidence of pituitary adenomas is approximately 5.1 cases per 100,000 population. The prevalence of clinically significant pituitary adenomas is estimated at 89.1 cases per 100,000, with a higher proportion of cases observed in female patients [12].

Brain tumors have a significant impact on both the quality of life and sleep of patients. Quality of sleep (QoS) is defined as “an individual’s satisfaction with all aspects of the sleep experience, which can be measured by the following variables: sleep efficiency, sleep latency, wakefulness after sleep onset, and by measures of sleep structure”. Sleep determinants include physiological (e.g., age, circadian rhythm, and body mass index), psychological (e.g., anxiety, depression), environmental factors (e.g., room temperature, use of electronic devices), and family/social obligations. Good QoS brings quantitative outcomes such as a sense of rest, normal reflexes, and positive relationships. In contrast, poor QoS contributes to poor health outcomes, with specific negative consequences such as fatigue, irritability, daytime dysfunction, delayed reactions, and increased consumption of caffeine and alcohol [13]. Literature reports that insomnia rates in cancer patients are almost three times higher than in the general population [14], with 30% to 60% of cancer patients experiencing symptoms of insomnia at least once during their treatment [14,15]. Studies that used validated sleep and insomnia questionnaires (Pittsburgh Sleep Quality Index, PSQI, and Insomnia Severity Index, ISI) exclusively in patients with primary brain tumors revealed that more than 50% of the patients experienced poor QoS, while 15% to 20% experienced moderate to severe symptoms of insomnia [16,17]. Furthermore, in a cross-sectional study [18], insomnia was associated with a decrease in QOL, which highlights the need for specific intervention to improve sleep and thus the daily life of patients with brain tumors. In addition, one of the most persistent and difficult symptoms, which has a negative impact on the daily activities and QOL of cancer patients, is fatigue [19]. Cancer-related fatigue (CRF) is defined as “a persistent, unusual and subjective feeling of physical, emotional and/or cognitive fatigue or exhaustion associated with cancer or its treatment that interferes with the person’s functioning” [20]. The prevalence of CRF is 52%, with statistically significant associations found between CRF and depression, insomnia, chemoradiotherapy, and the female gender. The pooled prevalence of CRF is higher in older patients and in patients undergoing chemotherapy and radiotherapy than in patients treated surgically [21].

Exercise may aid broadly in the management of glioma patients. More specifically, exercise enhances immune function by stimulating NK and T cells, thereby strengthening and promoting immune surveillance and tumor cell clearance. In addition, moderate exercise reduces chronic inflammation in the tumor microenvironment by promoting anti- inflammatory factors (IL-10, TGF-β) and lowering pro-inflammatory cytokines, which are responsible for activating tumor cell proliferation, migration, and metastasis, thereby potentially slowing glioma progression. Importantly, integrating exercise with chemotherapy or immunotherapy can improve therapeutic efficacy, lessen adverse effects, and contribute to a better quality of life for patients [22].

Different types of exercise have been investigated for their effect on CRF and QoS in cancer patients with varying results. A 6-week aerobic exercise intervention resulted in a statistically significant reduction in CRF among oncology patients [23], while Pilates interventions have also been associated with statistically significant improvements in CRF [24]. A 2017 meta-analysis reported that both aerobic and anaerobic exercise, when combined with psychological support, were more effective in alleviating CRF than pharmacological treatment [25]. In addition, it has been shown that combining a resistance exercise program with 12 weeks of aerobic training, performed at home, leads to faster recovery from CRF symptoms and improves QOL both during and after the end of radiotherapy [26]. Resistance training has also been shown to enhance QoS in prostate cancer patients [27], and systematic reviews have highlighted the effect of yoga on reducing sleep disturbances and improving QoS in breast cancer patients [28,29]. A meta-analysis comparing aerobic, resistance, and combined exercise modalities found significant reductions in CRF and insomnia across all approaches, with the greatest improvements observed for the mixed intervention of resistance and aerobic training. A previous review by Day et al., 2022 [30], evaluating both pharmacological and non-pharmacological interventions for fatigue in adults with primary brain tumors, identified three eligible trials, which investigated pharmacological agents and demonstrated uncertain effectiveness. Most exercise-based interventions were excluded due to the lack of high self-reported fatigue as a necessary inclusion criterion in that review. Consequently, there was insufficient evidence to support any specific exercise-based interventions for fatigue in this population, emphasizing an important gap in this area that the present exercise-focused review aims to address.

Exercise as a non-drug, low-cost, and easily accessible intervention has been reported to contribute positively to QOL, QoS, and fitness in cancer patients [31,32], with the effects being even greater in supervised exercise. Therefore, individual studies of the effect of exercise on specific symptoms, such as CRF [33] and sleep disturbances [34], in understudied clinical cancer populations are deemed important [33]. The existing literature focusing on patients with CNS tumors is significantly less compared to individuals with other types of cancer, such as breast or lung cancer. In neuro-oncology, patients with primary brain tumors are often excluded from research on sleep due to cognitive impairment, as reported in a review related to primary brain tumors [35]. However, sleep disturbance is a significant problem for these patients due to their vulnerabilities. They are at higher risk of cognitive impairment, and problems with sleep may exacerbate the neuropsychological deficits caused by the brain tumor and its treatment [36]. Based on this background, we conducted a systematic review complemented by a meta-analysis of clinical trials in glioma, which summarizes available research on the topic with the aim to evaluate the effect of exercise on QoS and CRF in patients with primary brain tumors. Aerobic exercise prominently enhances cardiorespiratory fitness and improves cardio-metabolic variables, whereas resistance exercise mainly promotes muscle strength and mass and alleviates muscle weakness, while both intervention styles could influence circadian rhythms and exert immunomodulatory effects. We hypothesized that the above types of interventions could both be beneficial and that additive effects may exist. It is thus possible that combined aerobic and resistance training interventions confer benefits regarding CRF, QoS, and sleep disturbances in patients diagnosed with primary brain tumors, whether single-modality exercise approaches succeed in doing so or not.

## 2. Materials and Methods

### 2.1. Study Design

This systematic review was conducted based on the PRISMA (Preferred Reporting Items for Systematic Reviews and Meta-Analyses Checklist) guidelines, and the process of selecting and identifying studies is illustrated in the corresponding flowchart [37]. The literature review protocol was registered at the International Prospective Register of Systematic Reviews (PROSPERO: CRD420251113132).

### 2.2. Sources of Information and Search Strategy

A comprehensive literature search was conducted across the following online databases: PubMed, Scopus, Cochrane Library, and CINAHL. The search covered all years from database inception. The search was conducted between June and July of 2025, and the data extraction was conducted in July 2025. Only studies published in English were considered. The search strategy combined Medical Subject Headings (MeSH) and free-text terms related to glioma, primary central nervous system tumors, exercise, cancer-related fatigue, sleep quality, and insomnia using Boolean operators (AND/OR). The studies were screened independently by two of the authors. Reference lists of included articles and relevant reviews were manually screened to identify additional eligible studies. The full electronic search strategy for each database is provided in Supplementary File S1.

### 2.3. Eligibility Criteria

The selection of studies included in this systematic review was based on predefined inclusion and exclusion criteria, structured according to the PICOS (Patient/Problem, Intervention or Treatment, Comparative Intervention, Outcomes) model [38]. More specifically, the eligibility criteria used to include studies in this systematic review are as follows: Population (P): Men and women over 18 years of age diagnosed with primary central nervous system (CNS) tumors. Consequently, studies that targeted patient samples with other cancer types (e.g., breast cancer) or included patients with CNS metastases from primary tumors of other organs were excluded. Intervention (I): Different types of exercise with adequate description of type, intensity, frequency, and duration of treatment. Comparator (C): Either a different type of exercise or a standard physiotherapy intervention. Outcomes (O): Objective, measurable outcomes for the variables of sleep quality, insomnia, and CRF. Study Design (S): Eligible study types included randomized controlled trials (RCTs), non-randomized controlled trials (non-RCTs), observational studies, feasibility studies, and case studies. Systematic reviews, meta-analyses, conference abstracts, and qualitative studies were excluded. Additional Inclusion Criteria: Studies had to be peer-reviewed articles and written in English, with full-text availability. No restrictions were applied consistently regarding the publication date. Additional Exclusion Criteria: Studies that did not isolate CNS oncology patients in their analyses were excluded. Studies that did not provide consistent numerical data were also excluded.

### 2.4. Article Selection Process

All retrieved references were imported into a common file, and duplicates were removed manually. The selection process was conducted in two phases. In the first phase, titles and abstracts were screened independently by two reviewers to assess potential eligibility. Full-text articles were evaluated in the second phase against the predefined inclusion and exclusion criteria. Studies were excluded if they involved cancer types other than primary CNS tumors, if they did not include an exercise intervention, if they failed to report outcomes separately for CNS patients, or were not retrievable in full text. When needed, discrepancies at the screening stages were resolved through consultation with a third reviewer. The detailed number of records identified, screened, included, and excluded at each stage is illustrated in the PRISMA flow diagram.

### 2.5. Evaluation of the Methodological Quality of Studies

The methodological quality of the included RCTs was assessed using the PEDro (Physiotherapy Evidence Database) scale [39]. This scale evaluates both the internal and external validity, and it includes 11 items. Each item is assigned one point when the item is rated as present (1 point) or absent (0 point), with a maximum score of 10; however, the first item, which pertains to external validity, is not included in the final score. As a result, the total possible score ranges from 0 to 10.

The JBI (Joanna Briggs Institute) critical appraisal scale for quasi-experimental studies was used to evaluate the non-RCTs. The scale is composed of 9 items, with each item scored as follows: yes, no, unclear, or not applicable. The questions test the internal validity, validity of statistical inferences, and quality of the study through specific safeguards that minimize the risk of bias. An empirical quality ranking based on adherence to the criteria at a rate of 100% is proposed; specifically, scores ≥ 70% are described as high quality, rates of 40–69% are assessed as moderate methodological quality, and rates < 40% as low [40].

To assess observational studies and case studies, we used the JBI checklist for analytical cross-sectional studies and the JBI checklist for case series accordingly. For the cross-sectional studies, the scale is composed of 8 items, while for the case series, the scale consists of 10 items, with each item scored as yes, no, unclear, or not applicable [41].

Two of the authors of this systematic review independently assessed the selected articles. In possible cases of disagreement regarding the scoring of the articles, the final decision was made after review by a third independent reviewer.

### 2.6. Evidence Quality

The GRADE (Grading of Recommendations, Assessment, Development and Evaluation) system was used to assess the quality of evidence from research findings. Quality was defined as the level of confidence in the assessment of the outcome to support a recommendation, considering factors such risk of bias in studies, inconsistency, imprecision, publication bias, indirect outcomes, and other factors that may affect the reliability of the evidence [42].

### 2.7. Data Extraction

Two independent authors performed the data extraction using a standardized extraction form. Relevant information from eligible studies was extracted, including the follow: study characteristics (author, year, study design, sample size, participant demographics like sex and age, and clinical features like tumor grade and therapies received); intervention details (exercise type, intensity, frequency, and duration); comparator characteristics for controlled studies; outcomes assessed (cancer-related fatigue, sleep quality, insomnia, sleepiness, and tools used); and main findings in measures of fatigue and sleep, including effect sizes or summary statistics (means, standard deviations, and *p*-values). Where data were incomplete or unclear, attempts were made to contact study authors. Any disagreements in data extraction were resolved by discussion with a third reviewer. Extracted data in more detailed tables can be found in Supplementary File S2.

### 2.8. Data Synthesis

We primarily utilized a narrative synthesis approach. Regarding glioma (≥2 RCTs available), we further performed meta-analyses to examine combined effects of controlled intervention studies via Comprehensive Meta-Analysis Software (CMA V2; Biostat, Inc., Englewood, NJ, USA). Where possible, extracted data were pooled to compare sleep and fatigue outcomes between intervention and control groups (3 studies evaluated sleep outcomes, and 6 studies evaluated fatigue outcomes in glioma patients). Parameters assessed were patient-reported measures provided via questionnaires for fatigue and sleep assessment (like BFI, FSS, VAS-F, FACIT-F, and PSQI and ISI, respectively). Effects sizes were calculated as Standardized Mean Difference (SMD) with a 95% confidence interval to account for different measurement scales used across studies. Where not directly available, reported means and standard deviations from pre- and post-intervention measurements were used to calculate change scores per group in each study for subsequent meta-analysis. Standard deviation of mean change was imputed according to Cochrane guidelines. In order to integrate one multi-arm study (Eisenhut et al. 2022 [43]) into the analysis of the overall effect of exercise on fatigue, we again implemented Cochrane guidelines to combine the two intervention groups of this study to create a single pairwise comparison. To identify differences between intervention and control, the random-effects model was employed to account for assumed variation across patient populations and study methodologies and provide more conservative and generalizable estimates of the overall effects [44]. Forest plots were generated to present the pooled effects. Pooled effect sizes were deemed statistically significant at *p* < 0.05. We used I^2^ values to determine the level of heterogeneity between studies (values < 25% indicate low, values of 50–75% moderate, and values > 75% high heterogeneity). Modality of treatment as potential source of heterogeneity was explored by limiting analysis to subsets of studies employing the same type of intervention (e.g., aerobic or resistance training).

## 3. Results

### 3.1. Article Selection

**Figure 1:**
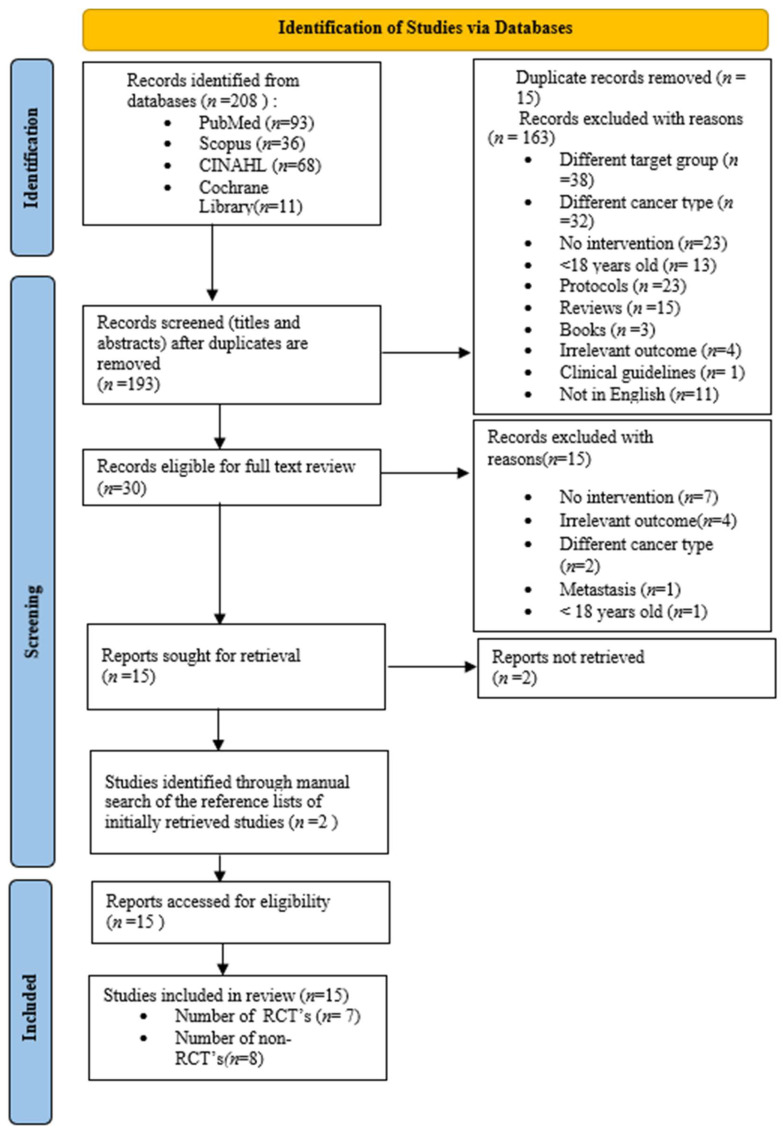
Flow chart diagram. Different target group = any population other than men and women over 18 years of age diagnosed with primary central nervous system (CNS) tumors.

Out of 208 initially identified articles in four databases, 15 were duplicates and thus removed and 163 were excluded at the title and abstract screening phase. The remaining 30 full-text articles were then reviewed considering the same predefined criteria, and 15 were further excluded, leaving a total of 15 studies sought for retrieval. An additional 2 studies from the 15 could not be retrieved, and 2 studies were added through manual search for a final total of 15. The studies selected in the brain tumor patients were the following: Gehrin et al., 2019 [45], Hansen et al., 2020 [46], Dülger et al., 2022 [47], Milbury et al., 2019 [48], Pieczyńska et al., 2023 [49], Jakkula et al., 2019 [50], Eisenhut et al., 2022 [43], Spencer et al., 2021 [51], Milbury et al., 2018 [52], Levin et al., 2015 [53], Colledge et al., 2017 [54], Capozzi et al., 2015 [55], Nowak et al., 2023 [56], Sandler et al., 2024 [57], and Miklja et al., 2022 [58]. From these, seven were RCTs [44,45,46,47,48,49,50] and eight were non-randomized [51,52,53,54,55,56,57,58] (non-RCTs, observational, and case–control). Reasons for the exclusion of studies at both screening phases can be found in Figure 1.

Detailed studies characteristics for RCT’s can be found in Table 1.

Detailed studies characteristics for non-RCT’s can be found in Table 2.

### 3.2. Methodological Quality of the Studies

The RCTs included in the current systematic review yield an average score of 5.7/10 according to the PEDro scale, as shown in Table 3. Both RCTs of high methodological quality and moderate methodological quality were included.

The mean score of the non-RCTs included in this review was 5.67/9 according to the JBI scale for quasi-experimental studies (Table 4). More specifically, five studies were classified as studies of moderate methodological quality, while the one study that scored 7/9 [57] was classified as studies of high methodological quality.

The JBI checklists for analytical cross-sectional studies and for case series were implemented to assess the observational study [58] and case series study [53], respectively. The score of the one observational study is 5/8 and of the one case series is 7/10. The tables and further assessment can be found in Supplementary File S3.

Assessment of the quality of evidence through the GRADE system revealed that, for the outcomes of CRF and QoS, there is moderate certainty of the evidence; Tables S7 and S8 for each outcome can be found in Supplementary File S4. For the outcome of CRF, seven RCTs were included; the five non- RCTs and one observational study introduced potential bias. Blinding concealments were unclear in three RCTs [48,49,50] and one non-RCT [51]. For the outcome of QoS, study designs included four non-RCTs and a case study, introducing a potential risk of bias. Blinding and allocation concealment were not adequately reported in three studies [52,53,56]. The absence of participant blinding in the included studies may introduce bias, as awareness of group allocation could potentially influence outcomes, particularly given that assessments were primarily based on self-reported questionnaires.

### 3.3. Study Characteristics

Detailed characteristics of the studies identified are presented in Table 1 and Table 2, including the type and grade of brain tumor, demographics of participants, type and other features of the intervention employed, and instruments used for evaluation, as well as main outcomes of interest. Below follows a brief description of the studies considered in this review.

#### 3.3.1. Randomized Controlled Trials

Eisenhut et al. (2022) [43] studied the impact of endurance and strength training on insomnia and CRF in high-grade glioma patients undergoing chemotherapy, radiotherapy, or both. Participants exercised in groups or individually, twice a week for six weeks (35–45 min/session). Endurance training included cycling or treadmill, while strength training focused on 3–5 sets of 10–15 reps for major muscle groups. The control group met twice per week to share experiences but did not exercise. Assessments took place at baseline, three weeks, and six weeks.

Gehring et al. (2019) [45] explored the effects of an aerobic exercise program on CRF and sleep in patients with Grade II/III glioma who had completed treatment at least six months earlier—unlike Eisenhut et al. (2022) [43], who included patients undergoing active treatment. The intervention group exercised three times per week, 20–45 min per session, at moderate to vigorous intensity (60–85% HRmax). The control group received a booklet with tips for staying active. Assessments were made at the end of the 6-month intervention.

Similarly to Gehring et al. (2019) [45], Milbury et al. (2019) [48] examined patients with glioma and assessed the impact of a dyadic yoga program on fatigue. Like Eisenhut et al. (2022) [43], this study focused on patients undergoing radiotherapy. Patients and their caregivers participated together in 45 min yoga sessions, two to three times per week, completing 12 sessions during treatment. The control group received standard care.

Dülger et al. (2022) [47] conducted a crossover RCT to compare yoga and a CE program in women with pituitary adenoma post-surgery. Participants completed six weeks of one intervention, followed by a two-week washout, then switched to the other. The CE program involved 30 min of aerobics and 30 min of resistance exercises, while the yoga group performed 60 min of yoga, each for three days a week. Sleep and CRF were evaluated at four time points.

Hansen et al. (2020) [46] examined the impact of physiotherapy and occupational rehabilitation in glioma patients undergoing active treatment, like studies [43,48]. The intervention group attended group-based sessions three times per week for six weeks, including aerobic exercise (75% HRR), resistance training (3 × 12 reps at 70–75% 1 RM), and 15 min of individual physiotherapy. The control group received standard care. Insomnia, CRF, and sleepiness were evaluated post-intervention.

Pieczyńska et al. (2023) [49] investigated the effects of augmented reality-based physical activity (AR) on CRF in high-grade glioma patients during radiotherapy, similar to studies [43,46,48]. Patients completed supervised 60 min sessions (5x/week, 70% HRmax) for one month, then continued at home using the augmented reality (AR)-Neuroforma program. Exercises included interactive games targeting mobility, strength, coordination, balance, and reaction speed. The control group continued normal activities. CRF was measured at baseline, after one month, and three months post-radiotherapy.

In contrast to other studies, Jakkula et al. (2019) [50] evaluated the effectiveness of Pilates group therapy. Participants had completed surgery, radiotherapy, or chemotherapy prior to the study, like previous studies [45,47]. The 12-week intervention included Pilates sessions three times per week for 60 min, featuring core-strengthening and flexibility exercises, followed by breathing and relaxation techniques. The control group followed the same home routine plus standard physical therapy. CRF was assessed pre- and post-intervention.

#### 3.3.2. Non-Randomized Controlled Trials

Spencer et al. (2021) [51] assessed an 18-month exercise program for high-grade glioma patients undergoing chemoradiotherapy, focusing on its effect on CRF. It included three groups: intervention, education, and control. Both intervention and education groups followed a 10-week program involving aerobic and strength training, along with self-reported diaries. Outcomes were measured at baseline, week 3, and week 10.

Capozzi et al. (2015) [55] implemented a 12-week group exercise program for patients with brain tumors. Each session, held once a week, totaled 9–10 strengthening exercises and 9–15 min of aerobic activity, along with a home exercise program twice a week. CRF and drowsiness were evaluated before and after sessions at weeks 1, 3, 6, 9, and 12.

Sandler et al. (2024) [57] studied patients with brain tumors, like those in Capozzi et al. (2015) [55], but delivered a supervised, individual exercise program. Their intervention was similar in structure to other studies [51,55] but lasted 18 weeks. All patients had completed 12–26 weeks of radiotherapy before starting. Like in an aforementioned study [51], participants did 150 min of moderate aerobic exercise and resistance training twice weekly. Outcomes were measured at baseline, mid-intervention, and at the end and included a six-month follow-up, unlike other studies.

Milbury et al. (2018) [52] examined the effect of a dual yoga program on QoS and CRF in patients with high-grade glioma and their caregivers during radiotherapy, which lasted 5–6 weeks. Yoga sessions were held 2–3 times per week. Unlike Sandler et al. (2024) [57], who studied patients after radiotherapy, they focused on exercise during radiotherapy. Outcomes were measured at baseline and after the intervention.

Collegde et al. (2017) [54], the only non-RCT focusing on meningioma patients, evaluated the effects of moderate aerobic exercise on subjective and objective sleep. The 12-week intervention program, similar in length to another study [55], involved 3–5 sessions per week at gradually increasing intensity. Participants included patients with aneurysmal subarachnoid hemorrhage, meningioma, and healthy controls. Sleep was assessed at baseline, post-intervention, and six months later, as in Sandler et al. (2024) [57].

Nowak et al. (2023) [56], like Spencer et al. (2021) [51], investigated high-grade glioma patients undergoing chemoradiotherapy using a CE program. CRF and QoS were evaluated pre- and post-intervention. The six-week program involved twice a week, one-hour outpatient sessions with moderate-intensity aerobic exercise and resistance training.

The CE training was also applied in the case study by Levin et al. (2016) [53]. They evaluated QoS in two patients with diffuse gliomas. Both completed two sessions per week for 12 weeks, including moderate-intensity aerobic exercise and resistance training, plus additional aerobic exercise at home. QoS was measured at baseline, six weeks, and after the intervention.

Miklja et al. (2022) [58] conducted an observational study on patients with high-grade glioma. They explored whether higher exercise tolerance was linked to lower fatigue and sleep disturbances. Patients completed a validated telephone survey to report on their exercise habits before and after diagnosis.

### 3.4. Results of Randomized Clinical Trials

#### 3.4.1. Outcomes for Cancer-Related Fatigue

All RCTs assessed CRF as an outcome following exercise interventions. A total of 245 oncology patients were enrolled, of whom 220 completed the sessions. There was a lack of consistency in the questionnaires used to measure fatigue, with only two studies [48,50] employing the same tool—the BFI. Most RCTs reported positive effects of exercise on CRF reduction, while only two studies [43,49] observed either a worsening or no change in CRF symptoms.

More specifically, two studies [46,47] reported a statistically significant reduction and improvement in fatigue, respectively, in the intervention group following a structured CE program. Statistically significant reductions in CRF were also observed in a Pilates intervention [50]; while using the same CRF assessment tool, marginally significant improvements were reported after a yoga program [48]. Gehring et al. (2019) [45] found a moderate effect size in CRF reduction following an aerobic exercise intervention. In contrast, Eisenhut et al. (2022) [43] reported negative outcomes, with no improvement in CRF after either aerobic or strength training interventions. Lastly, no significant changes in CRF following an AR-assisted exercise program comprising balance training were found, though a slight increase in perceived CRF was noted [49].

**Figure 2:**
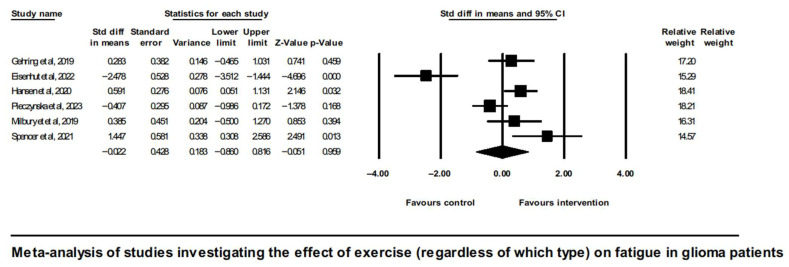
Forest plot of the meta-analysis of studies on glioma patients comparing exercise intervention group (regardless of which type) and control group with regard to fatigue outcomes [43,45,46,48,49,51].

To delineate the overall effect of exercise (irrespective of which type) on CRF in glioma patients, a meta-analysis of six eligible studies showed a neutral result (SMD = −0.022, [95% CI]—0.86 to 0.82, and *p* = 0.96), shown in Figure 2.

**Figure 3:**
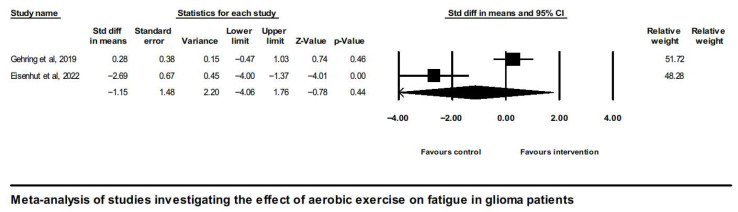
Forest plot of the meta-analysis of studies on glioma patients comparing aerobic exercise intervention group and control group with regard to fatigue outcomes [43,45].

Analysis of overall effects on fatigue revealed a high level of study heterogeneity (Q = 35.9, I^2^ = 86.1, and *p* = 0.00). Non-significant results were also found when limiting the analysis to aerobic exercise interventions [43,45] (Figure 3).

**Figure 4:**
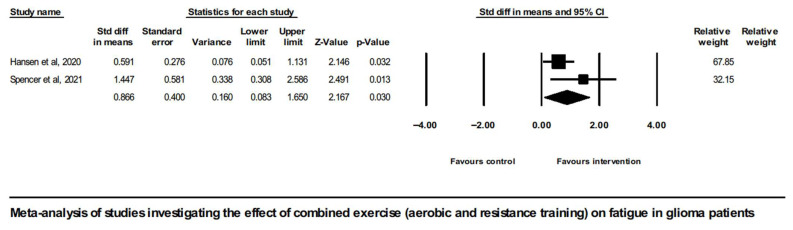
Forest plot of the meta-analysis of studies on glioma patients comparing combined exercise intervention group (aerobic and resistance training) and control group with regard to fatigue outcomes [46,51].

There was only one study involving a pure resistance training intervention group (Eisenhut et al. 2022 [43]); therefore, no meta-analysis was possible for this type of intervention. On the contrary, meta-analysis of the two studies implementing combined exercise intervention (aerobic and resistance training) did show a positive overall effect on CRF in glioma patients (SMD = 0.866, [95% CI] 0.083 to 1.650, and *p* = 0.03), shown in Figure 4.

It is noted that the study by Spencer et al., 2021 [51], did not follow randomization; nevertheless, it did involve a control group, and it did report enough statistics to be included alongside the RCTs in the meta-analysis of exercise effects on CRF in glioma. One of this study’s restrictions, however, was the imbalance in terms of the gender ratio between the intervention and control groups. A sensitivity analysis showed that either inclusion or exclusion of this study from the meta-analysis shown on Figure 2 did not change the overall effect, which in any case was not significant.

A further sensitivity analysis excluding the study by Gehring et al. (2019) [45], which differed from the rest of studies in the clinical characteristics of participants (excluding Grade IV and including less severe; Grade II post-treatment patients vs. Grade III and IV active chemo or radiation treatment patients included in all other studies) as well as had a longer duration of intervention (6 months vs. 4, 6, or 10 weeks in the rest of the studies), did not alter the overall neutral effect observed in Figure 2.

#### 3.4.2. Results for Sleep Parameters

Three RCTs [41,43,47] examined the effects of exercise interventions on QoS and insomnia in patients with brain tumors. Across the three trials, a total of 73 participants were enrolled, with 69 completing the interventions. Two studies [43,45] implemented aerobic exercise protocols, while one [47] utilized a CE program incorporating both physical exercise and yoga.

There was a moderate level of consistency in the assessment tools used across studies. Two trials [45,47] employed the PSQI to evaluate QoS, while one [43] used the ISI to assess insomnia. Aerobic exercise was found to be effective in reducing insomnia symptoms and to confer a moderate improvement in QOS by two studies [43,45]. Conversely, resistance training showed negative results with an increase in insomnia symptoms [43]. Yoga was reported to have greater improvements than CE but with notable enhancements also observed in the group receiving CE [47]. In addition, a statistically significant reduction in sleepiness was observed after the CE intervention among oncology patients [46].

**Figure 5:**
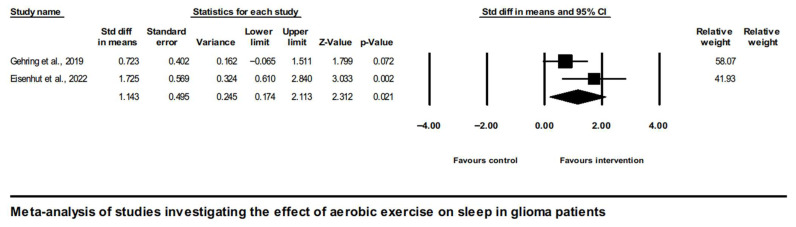
Forest plot of the meta-analysis of studies on glioma patients comparing aerobic exercise intervention group and control group with regard to sleep outcomes [43,45].

Consistently, pooling the effects of aerobic exercise interventions on sleep in glioma patients yielded a large positive effect (SMD = 1.14, [95% CI] 0.17 to 2.11, and *p* = 0.02), as shown in Figure 5.

The heterogeneity of studies was moderate (Q = 2.07, I^2^ = 51.7, and *p* = 0.15).

Resistance training, on the other hand, as well as combined exercise, each utilized by only one clinical trial (by Eisenhut et al., 2022 [43], in the former and by Hansen et al. [46] in the latter), did not warrant a meta-analysis. Lastly, yoga was reported to have greater improvements than CE but with notable enhancements also observed in the group receiving CE in pituitary adenoma patients [39].

### 3.5. Results of Non-Randomized Clinical Trials

#### 3.5.1. Results for Cancer-Related Fatigue

Of the eight non-RCTs, six investigated CRF as an outcome [51,52,55,56,57,58]). These non-RCTs included a total of 128 oncology patients, with 98 completing the interventions, fewer than the sample size observed in RCTs addressing fatigue. Notably, three of the studies used CRF assessment tools consistent with those used in the RCTs: Milbury et al. (2018) [52] employed the BFI, while studies [56,57] used the FACIT-F scale. Spencer et al. (2021) [51] utilized the VAS-F, Cappozi et al. (2015) [55] applied the ESAS, and Miklja et al. (2022) [58] assessed CRF using the PROMIS and Neuro-QoL v1.1 instruments.

The observational study described that low exercise endurance results in increased CRF symptoms, whereas high endurance characterized by moderate and high intensity exercise reduces CRF symptoms. In the majority of the remaining non-RCTs, there was a positive change in CRF after exercise intervention. More specifically, in the studies where CE was performed, there were positive results in three [51,55,57] and neutral results with no change in one [56]. No change was observed through the yoga intervention in one study [52].

#### 3.5.2. Outcomes for Sleep Parameters

Six out of eight non-RCTs reported outcomes related to QoS, insomnia, or sleepiness in a total of 115 oncology patients, with 92 completing the interventions. Three studies assessed QoS [52,53,56]), one focused on sleep disturbances [58], one insomnia [54], and one on sleepiness [55]. The PSQI was used by all three studies assessing QoS; one employed the ISI, the FEPS-II, and the Dysfunctional Sleep-Related Cognition scale [54]. The ESAS was used to evaluate sleepiness [55], while PROMIS and Neuro-QoL instruments assessed sleep disturbances [58].

Most studies reported improvements in sleep-related parameters. Among those using the same questionnaire to assess QoS (PSQI), the study that implemented a yoga intervention demonstrated clinically significant improvements [52]. In contrast, CE reported no significant changes in QoS and minimal improvement within two studies [53,56]. Aerobic exercise led to a reduction in insomnia symptoms and statistically significant decrease in sleepiness [54,55], respectively. Lastly, patients with higher exercise tolerance experienced fewer sleep disturbances [58].

## 4. Discussion

This systematic review explored the effects of various types of exercise on CRF, QoS, and insomnia in patients with CNS tumors. RCT studies evaluating exercise interventions in glioma patients demonstrated mixed results regarding cancer-related fatigue (CRF) and sleep outcomes. Some interventions such as combined exercise programs (aerobic and resistance training), yoga, and Pilates might lead to improvements in CRF, while resistance or aerobic training alone did not show benefits. Evidence from non-RCTs further supported the beneficial effects of combined exercise in reducing fatigue in these patients. In terms of sleep, aerobic exercise was associated with reduced insomnia and improved sleep quality, whereas resistance training showed negative or limited results. Yoga interventions also demonstrated clinically meaningful improvements in sleep quality, while combined exercise yielded positive changes for this outcome as well.

The significance of this topic lies in the current gap in the literature, as most existing studies focus on general oncology populations, with a primary emphasis on QoL rather than specific symptoms such as CRF or sleep disturbances. Although a previous Cochrane review [30] examined a broader range of interventions for fatigue in adults with primary brain tumors, it left out clinical trials on exercise interventions available at that time. The reason for the exclusion of these studies was the requirement of clinically significant levels of fatigue as criterion for inclusion in the meta-analysis to improve the clinical utility of the findings. Since our study focused only on exercise-based interventions and aimed to produce rather exploratory results identifying patterns useful for future, more rigorous research, we did not apply this strict inclusion criterion in our review. By utilizing information from all relevant studies on this subject, we addressed a gap not covered by the Cochrane review. Notably, no prior systematic review has examined the impact of exercise and physical interventions on these outcomes specifically in CNS oncology patients. The closest relevant work is by Sandler et al. (2021) [59], which primarily aimed to describe physical activity levels following a diagnosis of primary CNS cancer. While it also investigated the relationship between physical activity and health outcomes, as well as the effects of exercise participation, it did not provide data on the impact of specific exercise interventions on CRF or QoS. This limitation is largely due to the scarcity of both RCTs and non-randomized studies targeting this specific patient population.

Our study reviewed available clinical evidence on the subject, and although meta-analysis showed a non-significant overall effect of exercise in general on fatigue in glioma patients, further focused exploration did show a significant positive effect of combined exercise (aerobic and resistance training) on fatigue in this patient population. One study reported worsening fatigue with either aerobic-only or strengthening-only exercise when exercise was performed concurrently with anti-cancer therapy [43] in patients with advanced Grade III and IV disease. Exercise intensity in the mentioned study of Eisenhut et al. did not, however, markedly differ from CE studies, as judged by targeted HRmax for aerobic training or the Borg Scale equivalent (percentage of repetition maximum) for resistance training employed by other studies, such as the one by Hansen et al. [46], so that discrepancies cannot be readily attributed to one factor.

Exercise has been shown to modulate the immune response in patients with CNS tumors by enhancing cytotoxic T cell and NK cell activity, while reducing immunosuppressive populations such as Tregs and MDSCs, thus counteracting the tumor-permissive microenvironment [60]. Furthermore, exercise-derived myokines such as IL-7, IL-15, and IL-6 act systemically, promoting T cell and NK cell survival while simultaneously exerting anti-inflammatory effects that may mitigate cancer-related cachexia and support immune competence. Furthermore, mechanisms include normalization of the blood–brain barrier, reduction in hypoxia, and improved perfusion, which can facilitate drug delivery and enhance the efficacy of chemotherapy and immunotherapy [61].

Studies have highlighted the pathophysiology between exercise, sleep, and fatigue associated with cancer. These parameters interact through complex and bidirectional interactions involving numerous physiological and psychological pathways. Exercise has been found to affect body temperature through a rapid reduction in core temperature, promoting sleep onset and entry into the deeper stages of sleep. After exercise-induced hyperthermia, the thermoregulatory mechanism is activated, reducing body temperature through peripheral vasodilation. There is insufficient evidence in the literature to demonstrate direct improvement in QoS and quantity through changes in heart rate and autonomic activity after exercise [62]. Although no studies have directly examined circadian rhythm modulation via exercise in CNS tumor patients, preliminary evidence from broader oncology populations indicates that exercise may significantly benefit the circadian rhythm by entraining it [63]. In addition, exercise reduces CRF through anti-inflammatory and neurohormonal mechanisms. More specifically, studies have shown that it reduces pro-inflammatory cytokine levels, as well as regulates the hypothalamic–pituitary–adrenal axis function, preventing cortisol hypersecretion and reducing the feeling of fatigue [64,65]. Finally, evidence suggests that it increases mitochondrial function, improving energy efficiency and reducing the feeling of exhaustion [66].

A systematic review demonstrated statistically significant improvements in CRF following exercise interventions both during and after cancer treatment [67]. Similarly, a meta-analysis [68] found no significant differences in CRF outcomes between breast cancer patients who exercised during or after chemo-radiotherapy, supporting the overall efficacy of exercise.

A meta-analysis on exercise effectiveness in colorectal cancer patients concluded that the benefits of exercise are evident regardless of supervision level or timing relative to surgery or chemotherapy [69]. In the present review, the majority of both RCTs and non-RCTs involved supervised exercise, with only one RCT [45] being unsupervised. Despite this, the majority of the studies showed improvements in the outcomes of interest, across both supervised and unsupervised interventions. Given that only one study was explicitly unsupervised and two studies [47,50] did not specify the supervision type, findings of the present systematic review do not contradict those that reinforce the general effectiveness of exercise in oncological settings independent of supervision [69].

Our results are in line with reports by Singh et al. (2020) [69], in which according to whom the effect size for improvements in QOL and CRF was greater when the intervention involved CE. Similarly, Dong et al. (2023) [70] identified CE as the most effective exercise modality for cancer patients during treatment, with statistically significant improvements in CRF also observed after treatment completion. Among neuro-oncological studies examined in the present review, significant reductions in fatigue mediated by combined exercise were reported by three studies: two on glioma and one on pituitary adenoma [46,47,51]. Furthermore, improvements in CRF were observed post-treatment, lasting up to six months [57]. These findings appear to be consistent with a meta-analysis, which demonstrated a statistically significant reduction in CRF among oncology (breast cancer) patients undergoing chemotherapy and radiotherapy through CE interventions [71].

Although preliminary, findings of the current study indicate that CE may have a positive effect on reducing fatigue, regardless of the intervention duration. Specifically, two studies [46,47] implemented six-week CE programs, one conducted a ten-week program [51], and one examined an eighteen-week intervention [57]. Statistically significant improvements in fatigue symptoms were observed in the shorter six-week interventions, while the longer eighteen-week program demonstrated positive effects extending up to the six-month post-intervention follow-up.

Aerobic exercise has been shown to significantly reduce CRF, particularly in breast cancer patients, as demonstrated in a meta-analysis [68]. Similarly, Cramp and Byron (2012) [67] reported statistically significant benefits from aerobic training, whereas resistance training and low-intensity interventions did not yield comparable effects. Li et al. (2023) [28] further supported the effectiveness of aerobic exercise, identifying both short- and long-term fatigue improvements in patients with cancer compared to control groups. In the current systematic review, aerobic exercise was individually assessed in two RCTs. Of these, one [45] reported a moderate positive effect on CRF, while the other [43] found a worsening of CRF. Meta-analysis of both RCTs yielded a non-significant overall effect of aerobic exercise on CRF. A key difference between the studies was intervention duration, six months in Gehring et al. [45] versus six weeks in Eisenhut et al. [43], a factor that may have contributed to the contrasting outcomes.

A systematic review [72] demonstrated that resistance exercise interventions significantly improved CRF in breast cancer patients, with benefits observed regardless of whether anti-cancer therapy was administered during or after the intervention. Similarly, Li et al. (2023) [28] found that resistance training programs shorter than twelve weeks led to reductions in fatigue among gastrointestinal cancer patients compared to control groups. In contrast with these results, resistance exercise in the present review was examined by only one study, which reported a worsening of CRF. As only one RCT is available on resistance training in glioma patients, evidence is limited to allow for a definite conclusion on either a detrimental or beneficial effect of resistance training.

The forest plot and Q- and I-statistics indicated high heterogeneity between studies examining fatigue in glioma patients. Discrepancies between studies might be due to a variety of factors such as patient characteristics (age, gender, glioma grade, timing of tumor therapy, and neurological deficits), characteristics of the intervention (type, duration, frequency, and intensity of exercise), time interval from exercise program completion to evaluation, etc.

The statistically significant reduction in CRF following Pilates reported by Jakkula et al. (2019) [50] is supported by Boing et al. (2023) [73], who found that a longer-duration Mat Pilates program produced significant improvements in CRF in breast cancer patients both immediately post-intervention and at six- and twelve-month follow-ups. Moreover, a meta-analysis [74] demonstrated that yoga effectively reduces CRF in breast cancer patients. Similarly, Ma, Li, and Chan (2025) [75] reported, in a systematic review, statistically significant reductions in CRF symptoms during chemotherapy and radiotherapy across multiple cancer types following yoga practice. In contrast, results obtained from patients with high-grade glioma do not show statistically significant CRF changes after yoga, but only marginal improvements or no change was observed in two six-week studies [48,52]

Regarding our results on the effect of different types of exercise on QoS and insomnia, it is shown that exercise can lead to an improvement in the respective parameters. More specifically, it is noted that yoga brings clinically significant improvements in QoS [47,52]. In a systematic review examining the effect of yoga on breast cancer patients, there was an improvement in CRF and sleep disturbances with yoga compared to no intervention [29]. However, there was significant heterogeneity in the studies, with small and moderate size differences between groups that, although statistically significant, were not clinically important. Furthermore, results referred only to the short-term effect of yoga. Zhu et al. (2023) [76], in a subsequent meta-analysis, similarly showed significant improvement in sleep at the end of the yoga intervention, but with non-statistically significant differences at the first month, three–four months, and six months post-intervention.

With the use of aerobic exercise, an improvement in insomnia was observed [43,54] both after implementation of the intervention for six weeks and after twelve weeks. There was also a moderate positive effect on QoS after implementing a six-month aerobic exercise program [44]. Data derived from the meta-analysis of the RCTs in this review support large positive effects of aerobic exercise on QoS and insomnia in glioma patients. The sleepiness parameter improved to a statistically significant level with both CE intervention as well as solely aerobic exercise intervention [46,55].

CE has been shown to significantly improve QoS in cancer survivors, as evidenced by a meta-analysis using the PSQI questionnaire conducted by Gururaj et al. (2024) [63]. Maric et al. (2024) [32] reported benefits of both resistance exercises and CE on insomnia and sleep disturbances across various cancer types. However, resistance exercise alone did not significantly improve QoS, whereas CE did. In the present review, CE demonstrated some encouraging results, as two studies [46,52] showed significant improvements in QoS and little change, respectively, whereas one [46] showed no significant changes in sleep quality. Moreover, evidence from one individual study demonstrated that resistance training actually worsened insomnia symptoms [43].

Notably, aerobic exercise was associated with improvements in insomnia following both six- and twelve-week interventions [43,54], and also, a six-month aerobic program produced moderate positive effects on QoS [45]. Moreover, sleepiness significantly improved after both CE and aerobic exercise interventions [46,55]. A systematic review in a diverse cancer population found that short- and long-term aerobic training reduced insomnia symptoms, but neither intervention type produced statistically significant changes in insomnia severity [28]

### Strengths, Limitations, and Future Recommendations

This systematic review examined a diverse range of exercise interventions targeting CRF and/or sleep, comprising but not limited to Pilates, resistance exercise, and AR. Research on these exercise types and their impact on fatigue and sleep remains limited, in particular, in the clinical population of patients with cerebral neoplasms. Paradigms from other oncologic populations indicate that there is an encouraging therapeutic potential. Su et al. (2024) [77] explored virtual reality, AR and occupational therapy in patients with various cancers, reporting significant improvements in fatigue and sleep patterns. Similarly, Song et al. (2025) [78] identified walking as the most effective exercise for improving sleep disturbances in breast cancer survivors, with Pilates showing notable benefits. Nakano et al. (2018) [79], in a meta-analysis of classic modalities, compared aerobic exercise, resistance training, and CE in oncology patients, finding statistically significant reductions in both fatigue and insomnia across all exercise types, with greater improvements observed for CE programs.

The strengths of the current systematic review lie in the presentation of pooled knowledge, through inclusion of both RCTs and non-RCTs on a topic not previously addressed by similar reviews. Exclusion of non-RCTs might deprive a systematic review of valuable insights into a subject. It is important to emphasize that the retrieved studies on which the results of this systematic review were based were published within the last decade, which enhances the value and reliability of the findings. The analysis of data from recent studies reflects improved and modern methodological approaches and highlights the management of CRF and QoS in CNS cancer patients as an emerging field of increasing clinical interest. A high completion rate of interventions (392 out of 448 participants) adds to the reliability of the results. Finally, the involvement of two independent reviewers and a third, when necessary, ensures an unbiased assessment of the methodological quality and reduced bias.

The quality of most available studies for the outcome of CRF and QoS is moderate. These are mainly due to the uncertainty of blinding and allocation concealment and limited precision. However, the results of this systematic review on the effects of each of the various exercise interventions on CRF and QoS are based on single or only few studies, limiting robustness and comparability between exercise types. Additionally, most QoS and insomnia outcomes rely on subjective self-reported measures, with only one study using an objective sleep quality assessment [54].

The absence of objective sleep measurements—such as SOL, sleep efficiency, REM latency, and nighttime awakenings—limits study reliability and may introduce bias. Methods like actigraphy and polysomnography provide validated, quantifiable data and are not affected by recall bias or reporting variability, thus enhancing reliability and internal validity. While subjective assessments remain valuable for capturing patients’ perceived sleep quality, objective methods allow for greater accuracy, detection of subtle physiological changes, and comparability across studies [80,81]. As studies examined in this review focusing on CNS tumor patients rely predominantly on subjective assessments, the need for future trials to incorporate objective sleep measures is underscored. The small number of studies, despite some of them being of high or at least moderate methodological quality, restricts comprehensive analysis, especially by CNS tumor type or glioma grade, and also the analysis of possible moderator effects. Larger sample sizes are essential to enhance the reliability and generalizability of findings. Increasing sample sizes would strengthen the robustness of conclusions regarding the effects of interventions on fatigue and sleep outcomes and aid in the formulation of clinical guidelines. Variability in glioma grade (II, III, and IV) and treatment status among patients may have influenced levels of CRF and sleep disturbances, affecting outcomes. In particular, studies on interventions in patients with benign brain tumors were even scarcer than those in glioma patients and were presented in an isolated narrative manner. Due to the limited number of studies and the small sample sizes for tumor types other than glioma, we were thus unable to perform analyses by tumor type or treatment phase. This presents a meaningful limitation and thus highlights the need for future research to explore potential differences in exercise effects across tumor types and treatment stages. Given these limitations, more high-quality RCTs are needed to support future systematic reviews and enable more extensive meta-analyses. Future research should incorporate objective sleep measures and explore under-studied interventions like Pilates and virtual/augmented reality. Future studies on possibly additive effects of combined exercise interventions need to be well-controlled including an aerobic-only or resistance-only control group. Long-term follow-up at 6 and 12 months is also essential to assess the lasting effects on fatigue, QoS, and insomnia in CNS cancer patients. As shown in most of the studies, improvement in these parameters parallel the improvements in physical fitness, depression, and overall quality of life of patients with this difficult disease. Interestingly, a study focusing on lifestyle interventions by Rooney et al., 2022 [82], provides valuable insights into combined lifestyle approaches for QoL, insomnia, and CRF in CNS tumor patients. The positive results of this study emphasize opportunities for future research on multi-component interventions alongside exercise-specific approaches.

In light of promising new advancements in medical therapy for glioma, such as fluorescence-guided neurosurgery or different immunotherapies and their broader implementation in the care of glioma patients, it is necessary to continue research efforts regarding exercise in brain tumor patients in order to be able to provide clinical practice guidelines.

## 5. Conclusions

Considering the above-mentioned limitations, mainly the small number of studies and small number of participants in each study, the findings of this review of studies investigating the effect of exercise on fatigue and sleep in CNS tumor patients are regarded as exploratory, providing preliminary insights into an emerging subject. In summary, CE incorporating moderate-intensity aerobic and resistance training 2–3x/week could be an effective intervention in reducing CRF in patients with glioma. Other types of exercise that might be beneficial in terms of fatigue reduction that are nevertheless based on individual reports only are yoga and Pilates. Although available evidence is not enough to provide clinical guidance, supervised aerobic exercise 2–3x/week could improve QoS and insomnia in these populations. Lastly, single reports suggest that yoga and CE may also be beneficial regarding sleep parameters.

## Figures and Tables

**Table 1:** RCT studies characteristics.

Source	Study Subjects	Sample Size (N)	Mean Age	Sex (% Males)	Intervention	Parameters	Comparison	Outcome	Results
Eisenhut et al., 2022 [43]	Patient glioma Grade III and IV All patients underwent postoperative treatment: CT and/or RT	N = 29 ETG = 10 STG = 11 ACG = 8	ETG = 49.1 (±13.14) STG = 54.6 (±13.45) ACG = 53.0 (±10.78)	Not mentioned	ETG: ergometric bicycle or treadmill STG: structured and standardized workout to strengthen all major muscle groups such as weightlifting and resistance exercises with appropriated devices	Frequency: 2x/week Intensity: Borg RPE scale 11–14 (aerobic endurance) Intensity: Borg RPE scale 11–15 (resistance) Session Time: 35–45 min (aerobic) 30–45 (resistance Duration: 6 weeks ETG Mode: Frequency: 2x/week Session Time: 35–45 min Intensity: Borg RPE scale 11–14 Duration: 6 weeks	2x/week in supervised groups, 30–45 min per session, for 6 consecutive weeks	Insomnia (ISI), fatigue (FSS)	Fatigue increased in STG and ACG but decreased in ETG; insomnia scores decreased in ETG and ACG but increased in STG
Gehrin et al., 2019 [45]	Patients with astrocytoma, oligodendroglioma, and oligoastrocytoma Grade II and III	N = 32 (IG = 21, CG = 11)	IG: 49.2 (±8.9), CG: 48.0 (±11.9)	IG: 9 (43%), CG: 5 (45%)	Home-based aerobic exercise, remotely supervised	Frequency: 3x/week Session Time: 20–40 min Intensity: 60–85% HRmax Duration: 6 months	Leaflet with general lifestyle advice to maintain physical activity	Fatigue (MFI), sleep quality, (PSQI)	Moderate effect size of improvement in IG for sleep and fatigue
Hansen et al., 2020 [46]	Patients with glioma: Grade II-IV Treatments included: surgery, RT, and CT; surgery and CT; surgery and RT; other	N = 64 (IG = 32, CG = 32)	IG = 56.1 (±11.6), CG = 52.1 (±13.4)	IG = 26 (81%), CG = 18 (56%)	Supervised aerobic training and resistance training, plus 15 min of individualized physiotherapy	Frequency: 3x/week Session Time: 90 min Intensity: Aerobic 75% HRmax Resistance 70–75% Duration: 6 weeks	Standard rehabilitation care	Fatigue, insomnia, and sleepiness (EORTC QLQ-C30 and BN20)	IG had significantly reduced symptoms of fatigue but non-significant improvement in sleepiness
Dülger et al., 2022 [47]	Women with pituitary adenoma: prolactin-secreting adenoma, growth hormone-secreting adenoma, plurihormonal adenoma, follicle-stimulating hormone-secreting adenoma, and adrenocorticotropic hormone-secreting adenoma	G1 = 5, G2 = 5	G1 = 52.0 (±13.5), G2 = 41.8 (±14.0)	100% female	Aerobic exercise Resistance training Yoga	Frequency: 3x/week Session Time: 30 min (aerobic and resistance)/60 min (yoga) Intensity: Aerobic 50–70% HRmax Resistance 70–75% Duration: 6 weeks	Group 1: Aerobic + resistance training for first 6 weeks, 2 week washout period, yoga for the next 6 weeks Group 2: Yoga first, 2-week washout, aerobic + resistance training second	Fatigue (FSS), sleep quality (PQSI)	After the yoga program, FACT-Br improved; decreases in FSS. After aerobic and resistance training, no significant decrease in FSS
Milbury et al., 2019 [48]	20 patient–caregiver dyads; patients with glioma: Grade II–IV; and all had undergone surgery and were receiving CT	N = 20 dyads	Patients: IG = 47.91 (±14.66) CG = 44.73 (± 12.23) Caregivers: IG = 52.36 (±16.00), CG = 48.27 (±11.88)	Patients: IG = 5 (50%), CG = 5 (50%) Caregivers: IG = 3 (30%), CG = 4 (40%)	Dyadic yoga during the RT	Frequency: 2–3 supervised sessions/week Session Time: 45 min/session Duration: 6 weeks	Standard care	Fatigue (BFI)	Clinically marginal but favorable fatigue outcomes for IG patients
Pieczyńska et al., 2023 [49]	Patients with Grade III and IV gliomas	N = 47	IG = 45.59 (±11.15), CG = 60 (±13.55)	IG = 14 (82.35%), CG = 9 (56.25%)	Moderate-intensity exercise using the Neuroforma system, under supervision	Frequency: 5x/week Session Time: 60 min x session Intensity: HRmax = 70%. Duration: 1 month	Regular daily activities	Fatigue (FACIT-F)	No significant changes over time in either group Levels: CG: The level of fatigue was similar at all three time points (baseline, after RT, and after 3 months) IG: Perceived fatigue increased but not significantly
Jakkula et al., 2019 [50]	Brain tumor survivors who had completed surgery, RT, and CT with or without ongoing hormone therapy	N = 30 (IG = 15, CG = 15)	18–65	Total = 21 (70%), IG = 13 (86.7%), and CG = 8 (53.3%)	Pilates	Frequency: 3x/week Session Time: 60 min Duration: 12 weeks	Conventional therapy 12 weeks	Fatigue (BFI), QoL (EORTC QLQ-C30)	IG: Statistically significant improvement in fatigue; CG: Also improved but less significantly

Ιntervention Group = IG, Control Group = CG, Radiotherapy = RT, Chemotherapy = CT, min = minutes, Brain-cancer-specific HRQL Questionnaire = QLQ-BN20, Functional Assessment of Chronic Illness Therapy—Fatigue = FACIT-F, European Organization for Research and Treatment of Cancer QOL Questionnaire—Core 30 = EORTC QLQ-C30, Brief Fatigue Inventory scale = BFI, Fatigue Severity Scale = FSS, Insomnia Severity Index = ISI, Pittsburgh Sleep Quality Index = PSQI, Endurance Training Group = ETG, Strength Training Group = STG, Active Control Group = ACG, Borg rating of perceived exertion scale = Borg RPE Scale, Heart Rate Maximum = HRmax, Functional Assessment of Cancer Therapy—Brain = FACT-Br.

**Table 2:** Other studies characteristics.

Source	Study Design	Study Subjects	Sample Size (N)	Mean Age	Sex (% Males)	Intervention	Parameters	Outcome	Results
Spencer et al., 2021 [51]	Feasibility Pilot Study	Patients with High-Grade Glioma Undergoing CT and RT	Ν = 17 IG = 7 CG = 8 EG = 2	IG: 51.4 (± 17.2) CG: 57.6 (± 18.4) EG: 67 (± 1.4)	IG = 6, CG = 3, and EG = 1	IG and EG completed aerobic and strength training	IG supervised/EG unsupervised (strength training): Frequency: 2x/week Session Time: 50 min Duration: 10 weeks Unsupervised IG and EG: Session Time: 150 min/week aerobic training Duration: 10 weeks	Fatigue (VAS-F) (EORTC QLQ-C30)	Fatigue levels: reduction in the IG, increase in the EG and CG; EORTC QLQ-C30, fatigue levels: reduction in the IG, increase in the EG and in the CG
Milbury et al., 2018 [52]	Pilot Study	Patients with High-Grade Glioma (Grade IV) Undergoing RT	N = 10 Patients = 5 Caregivers = 5	Patients = 51.94 (± 20.20) Caregivers = 58.16 (± 10.15)	Patients = 1 (20) Caregivers = 2 (40)	Dyadic yoga intervention	Frequency: 2–3x/week Session Time: 60 min Duration: 5–6 week	Fatigue (BFI) sleep quality (PSQI)	Reduction in sleep disturbance
Levin et al., 2015 [53]	Case Study	Patient 1, Anaplastic Oligodendroglioma, Grade III Patient 2, Glioblastoma Multiforme	Ν = 2	Patient 1 = 58 Patient 2 = 61	Ν = 2 female patients	Aerobic exercise and resistance + home-based aerobic activities	Frequency: 2x/week Session Time: 20 min aerobic exercise and 40 min. of resistance training Intensity: moderate-to-vigorous Duration: 12 weeks	Sleep quality (PSQI)	Little change for sleep quality,; both participants consistently reported scores above 5 (=poor sleep quality)
Colledge et al., 2017 [54]	Exploratory Study	Patients with Meningioma, Survivors of Aneurysmal Subarachnoid Hemorrhage, and Healthy Controls; No RT	Ν = 48 G. aSAH = 15. G.M = 16 G.C = 17	G.M:59.3, (15.7) G. aSAH: 57.3 (8.9) G.C: 57.5, (12.4)	G. aSAH = 4 (27%) G.M = 8 (50%) G.C = 6 (35%)	Aerobic exercise: 1 supervised and 2–4 sessions unsupervised x week	Frequency: 3–5x/week Session Time: 30–40 min Intensity: Weeks 1–4: 55–65% of HRmax, Weeks 5–8: 65–75% of HRmax, Weeks 9–12: 75–85% of HRmax Duration: 12 weeks	Subjective sleep quality (ISI, FEPS II), objective sleep (overnight EEG)	Insomnia scores decreased across all groups and were consistently lower at all time points in the G.C.; G.M descriptively exhibited the shortest SOL compared to the other groups
Capozzi et al., 2015 [55]	Feasibility study	Patients with Oligodendroglioma, Glioblastoma, Astrocytoma, Oligoastrocytoma, Ependymoma, and Glioma; Grade II–IV; Treatment Status: Surgical Intervention, Surgery and RT, Surgery and CT, and Surgery, RT, and CT	Ν = 24 n = 14 completed the program n = 10 did not complete	Completers = 52.6 (±9.8) Non-completers = 51.6 (±14.6)	Completers = 9 (90.0%) Non-completers = 8 (80.0%)	Supervised aerobic and resistance training	Frequency: 3x/week Session Time: 3x [7–10 min, moderate-intensity aerobic exercise, 3 resistance exercises, 3–5 min, of moderate-intensity aerobic exercise Intensity: Duration: 12 weeks	Fatigue, daytime sleepiness (ESAS)	Reductions in fatigue and sleepiness
Nowak et al., 2023 [56]	Exploratory Study	Patients with Grade IV Glioma (Glioblastoma) Undergoing Concurrent CT-RT Participated in the Study	Ν = 17	Ν = 17, IG: 55.82 (±8.90) Ν = 13, and Non-exercise/withdrew: 47.61 (±14.10)	Ν = 5 (70.6%)	Supervised aerobic and resistance training	Frequency: 2x/week Session Time: 1 h Intensity: Aerobic training at ~60–85% of HRmax, 20–30 min., Resistance training at 60–85% of 1-repetition maximum Duration: 6 weeks	Fatigue (FACIT-F), sleep quality (PSQI)	No statistically significant changes in fatigue and sleep quality
Sandler et al., 2024 [57]	Feasibility study	Patients with Primary Brain Tumors: Glioblastoma, Astrocytoma, Oligodendroglioma, and Hemangiopericytoma Grade II-IV	Ν = 12	Μ.A = 51 (19)	Ν = 7 (58%)	Individualized aerobic and resistance training	Frequency: 2–3x/week Session Time: 10–60 min Intensity: Moderate Duration: 18 weeks	Fatigue (FACIT-F)	Improvements in FACIT-F score
Miklja et al., 2022 [58]	Observational Study	Patients Diagnosed with Glioma, High-Grade Glioma (Grade III and IV), and Low-Grade Glioma (Grade II)	N = 38	L.E.G = 53.6 H.E.G = 49	L.E.G = 5 (13.2%) H.E.G = 18 (47.4%)	Validated telephone-based survey		HRQOL	Significant improvements in fatigue and sleep disturbances for H.E.G.

Ιntervention Group = IG, Control Group = CG, Education Group = EG, Radiotherapy = RT, Chemotherapy = CT, min = minutes, group aneurysmal subarachnoid hemorrhage = G. aSAH, Group Meningioma = G.M, Brain-cancer-specific HRQL Questionnaire = QLQ-BN20, Functional Assessment of Chronic Illness Therapy—Fatigue = FACIT-F, European Organization for Research and Treatment of Cancer QOL Questionnaire—Core 30 = EORTC QLQ-C30, Visual Analog Fatigue Scale = VAS-F, Brief Fatigue Inventory scale = BFI, Electroencephalography = EEG, Sleep Onset Latency = SOL, Low-Endurance Group = L.E.G, High-Endurance Group = H.E.G., Heart Rate Maximum = HRmax, Edmonton Symptom Assessment System = ESAS, and Health-related Quality of Life = HRQOL.

**Table 3:** Score of PEDro (Physiotherapy Evidence Database) scale for RCTs.

Author	Eligibility Criteria	Random Allocation	Concealed Allocation	Baseline Comparability	Blind Participants	Blind Therapists	Adequate Follow-Up	Blind Assessors	Intention to Treat Analysis	Between-Group Comparisons	Point Estimates and Variability	Score *
Gehring et al. (2019) [45]	+	+	−	+	−	−	−	+	−	+	+	5/10
Hansen et al. (2020) [46]	+	+	+	−	−	−	+	+	+	+	+	7/10
Dügler et al. (2022) [47]	+	+	−	+	−	−	−	+	−	+	+	5/10
Milbury et al. (2019) [48]	+	+	+	+	−	−	−	+	+	+	+	6/10
Pieczyńska et al. (2023) [49]	+	+	−	+	−	−	−	−	−	+	+	4/10
Jakkula et al. (2019) [50]	+	+	−	−	−	−	−	+	+	+	+	5/10
Eisenhut et al. (2022) [43]	+	+	+	+	−	−	+	+	+	+	+	8/10

* Eligibility criteria does not count towards the final score.

**Table 4:** Score of JBI (Joanna Briggs Institute) scale for quasi-experimental studies.

Author	1	2	3	4	5	6	7	8	9	Score
Spencer et al. 2021 [51]	Y	N	N	Y	Y	Y	Y	N	Y	6/9
Milbury et al. 2018 [52]	Y	N	Y	Y	N	N	Y	N	Y	5/9
Collegde et al. 2017 [54]	Y	N	Y	Y	N	Y	Y	N	Y	6/9
Cappozi et al. 2015 [55]	Y	Y	Y	N	Y	N	Y	U	N	5/9
Nowak et al. 2023 [56]	Y	Y	Y	N	N	N	Y	Y	Y	6/9
Sandler et al. 2024 [57]	Y	Y	Y	N	Y	Y	Y	U	Y	7/9

1 = Is it clear in the study what is the “cause” and what is the “effect” (i.e., there is no confusion about which variable comes first)? 2 = Were the participants included in any similar comparisons? 3 = Were the participants included in any comparisons receiving similar treatment/care other than the exposure or intervention of interest? 4 = Was there a control group? 5 = Were there multiple measurements of the outcome both pre- and post-intervention/exposure? 6 = Was follow-up complete, and if not, were differences between groups in terms of their follow-up adequately described and analyzed? 7 = Were the outcomes of participants included in any comparisons measured in the same way? 8 = Were outcomes measured in a reliable way? 9 = Was appropriate statistical analysis used? Y = Yes, N = No, and U = Unclear.

## Data Availability

The data presented in this study are available upon request from the corresponding author.

## Supplementary Materials

The following supporting information can be downloaded at: https://www.mdpi.com/article/10.3390/neurosci7010014/s1, File S1: Search Strategy for electronic Databases; File S2: Data Extraction; File S3: Methodological quality of cross sectional and case series studies; File S4: Grade evaluation.

## Abbreviations

CNS	Central Nervous System
QOL	Quality Of Life
CE	Combined Exercise
WHO	World Health Organization
QOS	Quality Of Sleep
CRF	Cancer Related Fatigue
PQSI	Pittsburgh Sleep Quality Index
ISI	Insomnia Severity Index
PRISMA	Preferred Reporting Items for Systematic Reviews and Meta-Analyses Checklist
PROSPERO	International Prospective Register of Systematic Reviews
RCT	Randomized Control Trial
Non-RCT	Non Randomized Control Trial
PICO	Patient/Problem, Intervention or Treatment, Comparative Intervention, Outcomes
PEDRO	Physiotherapy Evidence Database
JBI	Joanna Briggs Institute
GRADE SMD	Grading of Recommendations, Assessment, Development and Evaluation Standardized Mean Difference
SOL	Sleep Onset Latency
