# Supplementary material for: An Investigation of the Effect of Exercise on Sleep Disturbances and Fatigue Symptoms in Patients Diagnosed with Primary Brain Tumors: A Systematic Review
Source: NeuroSci. 2026 Jan 15;7(1):14. doi: 10.3390/neurosci7010014 (PMC12821631; doi:10.3390/neurosci7010014)
Supplement: Supplementary file 1 [file neurosci-07-00014-s001.zip › Supplementary File S1.pdf]

Search Strategy for electronic Databases

2.4. Sources of information and search strategy

Search Strategy

Specifically, in the PubMed electronic database: the terms used in the search field were: "brain tumor" "exercise" "quality of sleep" "fatigue", "insomnia" "meningioma patients", "exercise intervention" "cancer related fatigue" "high grade glioma", "supervised aerobic exercise" "resistance exercise" "glioblastoma", "exercise" "high grade glioma" "fatigue", "primary brain cancer" "exercise intervention".

In the Scopus database: in the search field the words sleep AND quality/yoga AND therapy/fatigue/brain AND tumor, brain AND tumor/ Exercise/ Fatigue/ quality AND of AND sleep, Rehabilitation, Brain AND tumor, individualized AND exercise.

In addition, in the Cochrane Library search engine: in the Title Abstract Keyword field the words : "brain tumor" "yoga" "fatigue"

As for the CINAHL search engine: the terms were used: "pilates" "insomnia" "fatigue" "glioma".
