# Supplementary material for: An Investigation of the Effect of Exercise on Sleep Disturbances and Fatigue Symptoms in Patients Diagnosed with Primary Brain Tumors: A Systematic Review
Source: NeuroSci. 2026 Jan 15;7(1):14. doi: 10.3390/neurosci7010014 (PMC12821631; doi:10.3390/neurosci7010014)
Supplement: Supplementary file 1 [file neurosci-07-00014-s001.zip › Supplementary File S2.pdf]

2.7 Data Extraction

Table S1. RCT studies Characteristics.

Source	Study subjects	Sample size (N)	Mean age	Sex (% males)	Intervention	Comparison	Outcome	Assessment and Follow-up (months)	Results (with statistics)
Eisenhut et al, 2022 [43]	Patient Glioma Grade III: ETG: n = 2 STG: n = 4 ACG: n = 1 Glioma Grade IV: ETG: n = 8 STG: n = 7 ACG: n = 7 *Su*rgical Intervention (Number of participants per group: ETG, STG, ACG) No intervention: 0, 0, 1 Biopsy: 1, 1, 2 Partial resection: 3, 3, 1 Total resection: 6, 7, 4 All patients underwent postoperative treatment: chemotherapy and/or radiotherapy. Type of Oncological Treatment (n: ETG, STG, ACG) Combined therapy (chemo + radiotherapy): 10, 10, 7 Radiotherapy only: 0, 1, 1	N = 29 ETG = 10 STG = 11 ACG = 8	ETG = 49.1 ± 13.14 STG = 54.6 ± 13.45 ACG = 53.0 ± 10.78	Not mentioned	ETG Mode: Ergometric bicycle or treadmill Intensity: Borg RPE scale 11–14 (aerobic endurance) Frequency: 2x/week for 6 weeks Duration: 35–45 minutes per session STG Intensity: Borg RPE scale 11–15 (resistance) Frequency & Duration: 2x/week in supervised groups, 30–45 minutes per session, for 6 consecutive weeks	2x/week in supervised groups, 30–45 minutes per session, for 6 consecutive weeks. Participants did not engage in any structured physical activity during the study period	Insomnia – ISI Fatigue – FSS	Baseline, 3 weeks and 6 weeks	Fatigue increased in STG and ACG but decreased in ETG. Insomnia scores decreased in ETG and ACG, but increased in STG ANOVA (Time × Group Interaction) (F, Partial η^2) Fatigue=0.75 0.028 [S], 8.96*** 0.408 [L] 7.56*** 0.368 [L] Insomnia = 1.59 0.057 [S] , 0.09 0.007 [S] , 8.84*** 0.405 [L] Cohens'd (ETG, STG, ACG)Fatigue = 0.715 [M] 0.355 [S] 1.305 L] Insomnia= 1.654 [L] 0.379 [S], 0.599 [M]

Gehrin et al., 2019 [45]	Patients with Astrocytoma (IG = 29%, CG = 46%),<br>Oligodendroglioma (IG = 57%, CG = 46%),<br>Oligoastrocytoma (IG = 14%, CG = 9%).<br>Grade II (IG = 71%, CG = 55%),<br>Grade III (IG = 29%, CG = 46%)	N = 32 (IG = 21, CG = 11)	IG: 49.2 (±8.9), CG: 48.0 (±11.9)	IG: 9 (43%), CG: 5 (45%)	Home-based aerobic exercise, 3x/week for 6 months, 60–85% HRmax, 20–40 min per session, remotely supervised.	Leaflet with general lifestyle advice to maintain physical activity.	Fatigue, sleep quality<br>Tools: MFI, PSQI.	Baseline, 6 months	Moderate effect size in IG for sleep and fatigue.<br>IG mean/median change:<br>PSQI: −0.33 [−0.66; 0.66],<br>MFI-GF: +0.78 [0.52; 1.17], MFI-PF: +0.52 [0.26; 1.56], MFI-RA: +0.49 [−0.09; 1.07], MFI-RM: +0.19 [−0.29; 0.68], MFI-MF: +0.63 [0.00; 1.00]
Hansen et al.,2020 [46]	Patients with glioma: Grade II (IG = 16%, CG = 19%),<br>Grade III (IG = 22%, CG = 9%),<br>Grade IV (IG = 63%, CG = 72%).<br>Treatments included: surgery, radiotherapy, and chemotherapy (IG = 72%, CG = 69%); surgery and chemotherapy (IG = 3%, CG = 3%);<br>surgery and radiotherapy (IG = 25%, CG = 16%);<br>other (IG = 0%, CG = 13%).	N = 64 (IG = 32, CG = 32)	IG = 56.1 (±11.6), CG = 52.1 (±13.4)	IG = 26 (81%), CG = 18 (56%)	Supervised program, 3x/week, 90-minute sessions. Aerobic training at 75% HRmax and resistance training (3 sets at 70–75% of 1RM), plus 15 minutes of individualized physiotherapy.	Standard rehabilitation care	Fatigue, Insomnia, sleepiness evaluated via EORTC QLQ-C30 and BN20 (Global Health Status/QoL and symptom scales).	Baseline, 6 weeks	IG showed significantly better scores in HRQoL , significantly reduced fatigue (β = −13.4, 95% CI [−26.00, −0.72], p = 0.04), and non-significant improvement in sleepiness (β = −10.8, 95% CI [−27.60, 6.06], p = 0.20).
Dülger et al., 2022 [47]	Women with pituitary adenomas (N = 10):<br>Prolactin-secreting adenoma (PRL): n = 2<br>Growth hormone-secreting adenoma (GH; acromegaly): n = 5<br>Plurihormonal adenoma: n = 1	G1 = 5, G2 = 5	G1 = 52.0 ± 13.5 years<br>G2 = 41.8 ± 14.0 years	100% female	Aerobic exercise: 3 consecutive days/week for 6 weeks, 30 min/day at 50–70% HRmax<br>Resistance training: same frequency and duration as above, with moderate intensity (50–70% HRmax)	**Group 1**: Aerobic + resistance training for first 6 weeks → 2-week washout period → yoga for the next 6 weeks	Assessment of fatigue, sleep quality.<br>PQSI, FSI.	At 4 period points:<br>T0: Baseline<br>T1: After first 6-week intervention<br>T2: Post-washout (2 weeks)<br>T3: After second 6-week intervention	After the yoga program, FACT-Br improved. After the intervention (T3, Group A+ST/.Yoga)<br>QOS= 5.20 ± 2.73, p=0.68/ 3.36 ± 1.79, p=0.31<br>Fatigue = 4.41 ± 1.34, p=0.06/ 4.33 ± 1.12, p= 0.09

	Follicle-stimulating hormone-secreting adenoma (FSH): n = 1<br>Adrenocorticotropic hormone-secreting adenoma (ACTH; Cushing's disease): n = 1 Postoperative duration (mean ± SD) Group 1 (G1): 6.8 ± 2.48 years<br>Group 2 (G2): 5.8 ± 1.30 years				Yoga: 3 consecutive days/week for 6 weeks, 60 min/day	**Group 2**: Yoga first → 2-week washout → aerobic + resistance training second			
Milbury et al.,2019 [48]	20 patient-caregiver dyads. Patients with glioma: Grade II (n=2), Grade III (n=2), Grade IV (n=16). All had undergone surgery and were receiving chemotherapy.	N = 20 dyads	Patients: IG = 47.91 ± 14.66 (range 27–75), CG = 44.73 ± 12.23 (range 24–61)<br>Caregivers: IG = 52.36 ± 16.00 (range 34–74), CG = 48.27 ± 11.88 (range 28–66)	Patients: IG = 5 (50%), CG = 5 (50%)<br>Caregivers: IG = 3 (30%), CG = 4 (40%)	2–3 supervised sessions/week, 45 minutes per session (total 12 sessions) of dyadic yoga during the radiotherapy phase.	Standard care	Fatigue, BFI	Basile, 6 weeks (end of radiotherapy)	Clinically marginal but favorable fatigue outcomes for IG patients: BFI score change (Least Squares Mean): IG = −0.88, CG = 0.07<br>F = 0.72, p = 0.41, Cohen's d = 0.28<br>QoL: IG = +3.60, CG = −3.99, F = 1.92, p = 0.19, d = 0.69
Pieczyńska et al.,2023 [49]	Patients with Grade III & IV gliomas	N= 47	IG = 45.59 ± 11.15, CG = 60 ± 13.55	IG = 14 (82.35%), CG = 9 (56.25%)	5 sessions x week, moderate-intensity exercise using the Neuroforma system, 60 minutes x session under supervision for 1 month, followed by a remote program at home using Neuroforma. HRmax = 70%.	Regular Daily Activities	Fatigue (FACIT-F)	Baseline, 1 month (post-radiotherapy), and 3 months follow-up	IG: FACIT-F Baseline: 42 (28–52)<br>After RT: 40 (28–52)<br>After 3 months: 33 (22–49)<br>p value; 0.068
Jakkula et al., 2019 [50]	Brain tumor survivors who had completed surgery, radiotherapy, and chemotherapy, with or	N = 30 (IG = 15, CG = 15)	18-65	Total = 21 (70%), IG = 13 (86.7%), CG = 8 (53.3%)	Pilates: 60 min/day, 3 times/week for 12 weeks. Home program included 15 min walking 3x/week for 12	Conventional therapy 12 weeks	Fatigue, QoL<br>Tools: BFI, EORTC QLQ-C30	Baseline, 12 weeks	IG: statistically significant improvement in fatigue CG also improved but less significantly.<br>IG; BFI pre: 4.44±1.04<br>post: 3.87 ± 1.01

		without ongoing hormone therapy				weeks plus breathing exercises.			$p < 0.001$

Table S2. Other Studies Characteristics.

Source	Study design	Study subjects	Sample size (N)	Mean age	Sex (% males)	Intervention	Outcome	Assessment and Follow-up (months)	Results (with statistics)
Spencer et al.,2021 [51]	Feasibility Pilot Study	Patients with high-grade glioma undergoing chemotherapy and radiotherapy	N=17 IG=7 CG=8 Education Group (EG) =2	IG: 51.4 ± 17.2 years CG: 57.6 ± 18.4 years EG: 67 ± 1.4 years	IG=6, CG=3 , EG=1	IG and EG completed 60-minute supervised session x week (progressively increased to 150 minutes of aerobic exercise and 2 strength training sessions per week) and filled out a self-reported exercise diary weekly for 10 weeks.	Fatigue (EORTC QLQ-C30,VAS-F)	At weeks 0, 3, and 10	According to VAS-F, fatigue levels changed as follows: −46% reduction in the IG, +500% increase in the EG, +26% increase in the CG. According to EORTC QLQ-C30, fatigue levels changed as follows: −29% reduction in the IG, +33% increase in the EG, +35% increase in the CG
Milbury et al., 2018 [52]	Pilot Study (Single Arm)	Patients with High-Grade Glioma (Grade IV) Undergoing Radiotherapy and Their Caregivers	N=10 Patients = 5 Caregivers=5	Patients= 51.94 ± 20.20 (20-69) Caregivers = 58.16 ± 10.15 (48-73)	Patients=1 (20) Caregivers = 2 (40)	Dyadic yoga intervention: 2-3 weekly sessions, 60 minutes each, delivered during the patients' 5–6 week course of radiotherapy, led by a certified yoga instructor.	Fatigue, sleep quality(BFI, PSQI)	Baseline and after 12 sessions, at 5-6 weeks.	Reduction in sleep disturbance. BFI Post-intervention: Mean = 1.51, SD = 1.64, $t = -0.03$, $p = 0.98$, $d = 0.02$. PSQI Post-intervention: Mean = 8.00, SD = 1.41, $t = -1.35$, $p = 0.10$, $d = 1.17$*
Levin et al., 2015 [53]	Case Study	Patient 1, anaplastic oligodendroglioma (WHO Grade III). Patient 2 glioblastoma multiforme.	N=2	Patient 1=58 Patient 2=61 years	N=2 female patients	12-week supervised exercise program,2 sessions x week. Session of 20 minutes moderate-to-vigorous aerobic exercise and 40 minutes of resistance and additional aerobic training, complemented by home-based aerobic activities. At least 150 minutes of aerobic exercise x week, excluding the resistance training sessions.	Sleep quality (PSQI)	Baseline, Week 6, and Week 12	Consistently high PSQI scores (>5) indicating poor sleep quality Patient 1 PSQI Start=16/ 6 weeks =17 /12 weeks=12 Patient 2 PSQI Start=12/ 6 weeks=14 /12 weeks=15

Colledge et al., 2017 [54]	Exploratory Study	16 patients with meningioma, 15 survivors of aneurysmal subarachnoid hemorrhage (aSAH) and 17 healthy controls. None were undergoing radiotherapy.	N= 48 G. aSAH= 15 .G.M =16 G.C=17	G.M: M.A=59,3, S.D= 15,7 G. aSAH: M.A = 57,3 S.D = 8,9 G.C: M.A =57,5, S.D = 12.4	G. aSAH=4 (27%) G.M =8 (50%) G.C=6 (35%)	12-week training program, 3–5 sessions x week of moderate aerobic exercise (30–40 minutes per session) Exercise intensity: Weeks 1–4: 55–65% of HRmax, Weeks 5–8: 65–75% of HRmax, Weeks 9–12: 75–85% of HRmax. One supervised session per week, and 2–4 sessions unsupervised	Subjective sleep quality: ISI, FEPS II Dysfunctional Sleep-Related Cognitions, Objective sleep: Overnight EEG recording using portable EEG device	Baseline, after 12 weeks, and at 6-month follow-up	Insomnia scores decreased across all groups and were consistently lower at all time points in the control group. The meningioma group descriptively exhibited the shortest SOL compared to the other groups. Effect sizes baseline to post-test, Cohen's d (G.aSAH, G.M , G.C) SOL (min): -0,360, 0,509,-0,076 Insomnia: -0,038, -0,178, 0,124
Capozzi et al., 2015 [55]	Feasibility study	Among 24 patients: 9 (37.5%) had oligodendroglioma, 7 (29.2%) had glioblastoma, 4 (16.7%) had astrocytoma, 2 (8.3%) had oligoastrocytoma, 1 (4.2%) had ependymoma, 1 (4.2%) had glioma (unspecified). Regarding tumor grade: 8 patients (33.3%) were Grade II, 7 (29.2%) were Grade III, 7 (29.2%) were Grade IV, 2 (8.3%) had unclear grading. Treatment status: 10 patients (41.7%) were undergoing current treatment, Surgical intervention only: 7 patients (29.2%), Surgery and radiotherapy: 2 patients (14.3%), Surgery and chemotherapy: 1 patient (4.2%), Surgery, radiotherapy, and chemotherapy: 14 patients (58.3%)	N=24 n=14 completed the program n=10 did not complete	Completers=52,6 ± 9,8 Non-completers =51,6 ± 14,6	Completers=9 (90.0%) non-completes = 8 (80.0%) Total=17 (70.8%)	12-week group-based exercise program, 1 supervised session per week. Session included: 7–10 minutes of moderate-intensity aerobic exercise, 3 resistance exercises. Followed by 3–5 minutes of moderate-intensity aerobic exercise This aerobic/resistance set was repeated 3 times, totaling: 9–10 strength exercises, 9–15 minutes of aerobic training, Plus a 7–10 minute warm-up Additionally, participants performed one home-based exercise session twice per week	Fatigue, daytime sleepiness (ESAS)	Weeks 1, 3, 6, 9, and 12 (pre-exercise assessment at each time point)	Reductions were observed in fatigue and sleepiness. Mean changes (10-point scale): Fatigue: −2.6 ± 2.4, p = 0.002, Sleepiness: −2.2 ± 2.4, p = 0.004

Nowak et al.,2023 [56]	Explorator y Study	17 patients with Grade IV glioma (glioblastoma) undergoing concurrent chemoradiotherapy participated in the study	N=17	N=17, Intervention group (completed exercise program) 55,82 ± 8,90 N=13, Non-exercise / withdrew group 47,61 ± 14,10	N=5 (70,6%)	6 weeks, 2 x week for 1 hour, supervised outpatient exercise facility, Aerobic training: Moderate to vigorous cardiovascular exercise (targeting ~60%–85% of HRmax) for 20–30 minutes, Resistance training: Moderate to high-intensity resistance exercises (60%–85% of 1-repetition maximum, 6–12 repetitions, 2–4 sets x exercise	Fatigue, sleep quality, FACIT-F , PSQI	Baseline and after6 weeks.	No statistically significant changes were observed in fatigue, sleep quality. FACIT-F pre-intervention= 39.79 ± 5.75 post-intervention=37.43 ± 6.25 p-value=0.281 PSQI pre-intervention=11.71 ± 6.2 post-intervention=11.21 ± 4.48 p-value=0.
Sandler et al.,2024 [57]	Feasibility study	Patients with primary brain tumors. Glioblastoma: 5 (42%), Astrocytoma: 3 (25%), Oligodendroglioma: 3 (25%), Hemangiopericytoma: 1 (8%) Disease Stage, n (%): Grade II: 3 (25%), Grade III: 4 (33%), Grade IV: 5 (42%), Treatment Received, n (%): Surgical resection: 11 (92%), Radiotherapy: 11 (92%)	N=12	M.A (S.D)= 51 (19)	N=7 (58%)	18 weeks of individualized, moderate-intensity aerobic and resistance training. Exercise dose was 150 minutes of moderate-intensity physical activity Resistance training 2–3 days per week. Session durations ranged from 10 to 60 minutes. Resistance training sessions included 1–4 sets of 5–10 repetitions per set across 2–8 exercises.	Fatigue (FACIT-F)	Baseline,week 9, week 18 and 6 months post-intervention	Improvements were observed in FACIT-F score (range 0–52): Mean change from baseline to post-intervention: 1.0 (95% CI: −2.7 to 4.8), p = 0.59, Mean change from post-intervention to follow-up: (95% CI: −2.3 to 4,3)
Miklja et al., 2022 [58]	Observati onal Study	Patients diagnosed with glioma, categorized by tumor grade and physical endurance level. High-Grade Glioma (Grade III & IV): Low endurance: 8 patients (21.1%), High endurance: 20 patients (52.6%), Low-Grade Glioma (Grade II): Low endurance: 3 patients (7.9%), High endurance: 7 patients (18.4%)	N= 38	Low Endurance group =53.6 High Endurance group =49	Low Enduranc e group = 5 (13.2%) High Enduranc e group = 18 (47.4%)	A validated telephone-based survey was conducted.	HRQOL assessments were conducted	No follow-up	Significant improvements were observed in the domains of fatigue and sleep disturbances in the high endurance group. Fatigue t-score: Low Endurance: 56.4, High Endurance: 49.3, p = 0.04. Sleep t-score: Low Endurance: 56.4, High Endurance: 48.7, p = 0.02

* Intervention Group= IG, Control Group= CG, Education Group=EG , minutes=min, Heart Rate maximum= HRmax, Group aneurysmal subarachnoid hemorrhage= G.aSAH, Group Meningioma=G.M, Multidimensional Fatigue Inventory =MFI, Brain-cancer specific HRQL Questionnaire= QLQ-BN20 , MOS Short- Form 36 = SF-36, Fatigue Severity

Index= FSI, A+ST= aerobic & strength training, Functional Cancer Therapy Assessment-Brain= FACT-Br, Functional Assessment of Chronic Illness Therapy—Fatigue =FACIT-F, Borg Rating of perceived exertion scale= Borg RPE Scale, Edmonton Symptom Assessment System= ESAS, Health related Quality of Life= HRQOL, European Organization for Research and Treatment of Cancer QOL Questionnaire–Core 30= EORTC QLQ-C30, Visual Analog Fatigue Scale= VAS-F, Brief fatigue inventory scale= BFI, , Fatigue Severity Scale =FSS, Sleep onset latency=SOL, Multidimensional Assessment of Fatigue=MAF, Endurance Training Group =ETG, Strength Training Group =STG, Active Control Group =ACG.
