# Supplementary material for: An Investigation of the Effect of Exercise on Sleep Disturbances and Fatigue Symptoms in Patients Diagnosed with Primary Brain Tumors: A Systematic Review
Source: NeuroSci. 2026 Jan 15;7(1):14. doi: 10.3390/neurosci7010014 (PMC12821631; doi:10.3390/neurosci7010014)
Supplement: Supplementary file 1 [file neurosci-07-00014-s001.zip › Supplementary File S3.pdf]

Methodological quality of cross sectional and case series studies

Table S5. JBI scale for Analytical Cross sectional studies.

**Author**	**Miklja et al. 2022 [58]**
Criteria	YES-NO-UNCLEAR
1	YES
2	YES
3	UNCLEAR
4	YES
5	YES
6	UNCLEAR
7	YES
8	NO
**Score**	**5/8**

1= 1. Inclusion criteria defined? 2. Subjects & setting described? 3. Exposure measured validly & reliably? 4. Standard criteria for outcome measurement? 5. Confounders identified? 6. Strategies to deal with confounding? 7. Outcomes measured validly & reliably? 8. Appropriate statistical analysis?

Criteria that were not fully met or deemed unclear were 3,6,8. Criterion 3, that addresses the reliability and validity of the ways the exposures were measured, was partially met because the exposure of exercise participation was measured using a self-reported questionnaire. Criterion 6 , which focused on the statement of the strategies to deal with confounding factors, was unclear due to the lack of mention of adjustment for potential confounding factors. Criterion 8, responds to the use of appropriate statistical analysis, was not met because they did not employ control for confounding variables.

Table S6. JBI checklist for case series.

**Author**	**Levin et al. 2015 [53]**
Criteria	YES-NO-UNCLEAR
1	YES
2	YES
3	YES
4	UNCLEAR
5	UNCLEAR
6	YES
7	YES
8	YES
9	UNCLEAR
10	YES
**Score**	**7/10**

1= 1. Inclusion criteria defined? 2= Condition measured in a reliable way? 3=Were valid methods used for identification of the condition for all participants included in the case series? 4=Did the case series have consecutive inclusion of participants? 5=Did the case series have complete inclusion of participants? 6=Was there clear reporting of the demographics of the participants in the study? 7=Was there clear reporting of clinical information of the participants? 8=Were the outcomes or follow up results of cases clearly reported? 9=Was there

clear reporting of the presenting site(s)/clinic(s) demographic information? 10=Was statistical analysis appropriate?

Criteria that were not fully met and deemed unclear were 4,5,9. Criterion 4 that addresses the consecutive inclusion of participants, was unclear due to the lack of information whether the two participants represented all consecutive eligible patients within a specific time frame. Criterion 5, which responds to the complete inclusion of the participants, was unclear, as an extension of criterion 4 because there is no information on the number of the participants that were initially screened or invited to take part. Lastly, for criterion 9, the study mentioned that the participants were recruited through a rehabilitation setting, but there were no further information about it (timeframe of recruitment, name or type of institution).
