# Supplementary material for: An Investigation of the Effect of Exercise on Sleep Disturbances and Fatigue Symptoms in Patients Diagnosed with Primary Brain Tumors: A Systematic Review
Source: NeuroSci. 2026 Jan 15;7(1):14. doi: 10.3390/neurosci7010014 (PMC12821631; doi:10.3390/neurosci7010014)
Supplement: Supplementary file 1 [file neurosci-07-00014-s001.zip › Supplementary File S4.pdf]

Table S7. GRADE evaluation – Outcome: Sleep Quality (QoS). Studies included: 5 (2 RCTs, 2 non-RCTs, 1 case study)

No. of studies	Study design	Risk of bias	Inconsistency	Indirectness	Imprecision	Other considerations	Impact	Certainty	Importance
5	2 RCTs, 2 non-RCTs, 1 case study	serious[a]	not serious	not serious	serious	none	Most studies showed moderate improvements in sleep quality following exercise interventions.	Moderate	Important

CI: confidence interval. Explanations:. [a] Study designs included non-RCTs and a case study, introducing high risk of bias. Blinding and allocation concealment were not adequately reported in three studies ([43], [47],[48]).

Table S8. GRADE evaluation– Outcome: Cancer-Related Fatigue (CRF). Studies included: 13 (7 RCTs, 5 non-RCTs, 1 observational study).

No. of studies	Study design	Risk of bias	Inconsistency	Indirectness	Imprecision	Other considerations	Impact	Certainty	Importance
13	7 RCTs, 5 non-RCTs, 1 observational	serious[a]	not serious	not serious	serious	large effect	Most studies reported improvements in CRF following exercise interventions, with moderate to large effects.	Moderate	Important

Explanations: [a]. Although 7 Randomized Controlled Trials were included, the 5 non-randomised controlled trials and 1 observational study introduced potential bias. Blinding concealment were unclear in 3 RCT, ([48], [49] ,[50] and one non-RCT [51].
